# Imaging Biomarkers of Oral Dysplasia and Carcinoma Measured with In Vivo Endoscopic Optical Coherence Tomography

**DOI:** 10.3390/cancers16152751

**Published:** 2024-08-02

**Authors:** Jeanie Malone, Chloe Hill, Adrian Tanskanen, Kelly Liu, Samson Ng, Calum MacAulay, Catherine F. Poh, Pierre M. Lane

**Affiliations:** 1 Department of Integrative Oncology, British Columbia Cancer Research Institute, 675 W 10th Ave., Vancouver, BC V5Z 1L3, Canadaplane@bccrc.ca (P.M.L.); 2 School of Biomedical Engineering, University of British Columbia, 251-2222 Health Sciences Mall, Vancouver, BC V6T 1Z3, Canada; 3 School of Engineering Science, Simon Fraser University, 8888 University Drive, Burnaby, BC V5A 1S6, Canada; 4 Department of Oral Biological and Medical Sciences, Faculty of Dentistry, University of British Columbia, 350-2194 Health Sciences Mall, Vancouver, BC V6T 1Z3, Canada; 5 Department of Pathology and Laboratory Medicine, University of British Columbia and Vancouver General Hospital, G227-2211 Wesbrook Mall, Vancouver, BC V6 T 1Z7, Canada

**Keywords:** optical coherence tomography, oral cancer, endoscopic imaging, cancer morphology, optical biopsy

## Abstract

**Simple Summary:**

Oral cancers are associated with high mortality in advanced stages. Early diagnosis is associated with better patient outcomes, but this is challenging to achieve as benign lesions look similar to lesions of concern, and multiple biopsies may be required to ensure the most pathologic tissue is sampled. This work leverages a previously developed endoscopic imaging system and deep learning segmentation tool to provide measurements of subsurface changes in the first few millimeters of oral tissue. We present seven quantitative features that allow for rapid examination of tissue, which we propose may be useful for biopsy site or treatment margin selection.

**Abstract:**

Optical coherence tomography is a noninvasive imaging technique that provides three-dimensional visualization of subsurface tissue structures. OCT has been proposed and explored in the literature as a tool to assess oral cancer status, select biopsy sites, or identify surgical margins. Our endoscopic OCT device can generate widefield (centimeters long) imaging of lesions at any location in the oral cavity—but it is challenging for raters to quantitatively assess and score large volumes of data. Leveraging a previously developed epithelial segmentation network, this work develops quantifiable biomarkers that provide direct measurements of tissue properties in three dimensions. We hypothesize that features related to morphology, tissue attenuation, and contrast between tissue layers will be able to provide a quantitative assessment of disease status (dysplasia through carcinoma). This work retrospectively assesses seven biomarkers on a lesion-contralateral matched OCT dataset of the lateral and ventral tongue (40 patients, 70 sites). Epithelial depth and loss of epithelial–stromal boundary visualization provide the strongest discrimination between disease states. The stroma optical attenuation coefficient provides a distinction between benign lesions from dysplasia and carcinoma. The stratification biomarkers visualize subsurface changes, which provides potential for future utility in biopsy site selection or treatment margin delineation.

## 1. Introduction

Oral squamous cell carcinoma (OSCC) accounts for almost all malignant tumors of the lip and oral cavity and is often detected in advanced stages associated with high mortality [1,2]. Early diagnosis is associated with better patient outcomes, but this is challenging to achieve. 

Oral cancers often progress from normal tissue, through grades of dysplasia, to carcinoma [3,4]. During progression, there are cellular changes in size, shape, and nuclear properties, as well as larger changes in tissue architecture [5]. The earliest dysplastic changes (mild dysplasia) are characterized by architectural changes and cellular atypia in the bottom third of the epithelium closest to the epithelial–stromal boundary. Moderate and severe dysplasia represent changes in the bottom two-thirds of the epithelium and more than two-thirds of the depth of the epithelium, respectively [4]. While mild and moderate dysplasia are reversible and are generally subject only to monitoring, severe dysplasia through carcinoma requires treatment.

If a biopsy is taken of the lesion, architectural and cytologic changes can be assessed histologically [6]. However, identifying the most pathologic site within a lesion is challenging and is further complicated by benign lesions that may have a similar appearance to occult lesions [7]. As such, multiple biopsies are often taken to avoid false negatives or underdiagnosis. Tools that allow for noninvasive monitoring of subsurface structures may reduce the need for biopsy in mild and moderate dysplasia and aid in biopsy site selection to improve diagnostic yield. Similarly, surgery is the primary treatment for OSCCs. Capturing all margins reduces recurrence, but excessive margins may have negative cosmetic or functional effects [8]. A tool that could improve margin delineation may improve cancer-free survival and minimize surgery-related morbidities.

Optical coherence tomography (OCT) is a noninvasive, label-free imaging technique that generates high-resolution imaging of subsurface structures at a limited depth [9]. OCT is an interferometric technique: images are produced by scanning a low-coherence beam of light across a sample and interfering with the collected backscattered light with a path length-matched reference beam. The lateral resolution of OCT is a function of the focusing optics; the axial (depth) resolution is a function of the light source. OCT devices can be tailored to their application: some non-contact scanning devices use supercontinuum light sources and objective lenses to achieve sub-micron resolutions [10], while fiber-optic-based endoscopic systems are optimized to access luminal organs at the cost of lower resolutions (~40 µm lateral) [11,12]. This flexibility has allowed OCT to be explored in many cancer imaging applications [13]. The geometry captured in OCT has been shown to correlate precisely with histology [14], and the intensity of OCT can be used to estimate the depth-resolved attenuation coefficient, providing insights into the optical properties of the sample [15]. 

OCT has been previously explored in oral cancer applications [16]. Studies have assessed ex vivo tumor tissue for diagnostic utility in oral precancerous or potentially malignant lesions [17,18,19,20,21,22,23]. In vivo oral OCT has primarily been conducted with hand-held galvanometer scanning devices [24,25,26,27,28,29]; we have previously shown endoscopic OCT of the oral cavity acquired with rotary-pullback catheters [30]. OCT can visualize keratin, epithelial, and subepithelial layers, the presence or absence of the epithelial–stromal boundary, and microanatomical features such as vasculature, salivary ducts, or rete pegs. 

OCT in oral cancer applications is an area of active research, and there is currently no consensus on diagnostic criteria. Approaches from qualitative classification systems [26] to quantitative measurements of epithelial thickness [17,28,31,32], optical attenuation [33], or texture features [22] have been explored. Epithelial thickness has been measured in vivo for different sites across the oral cavity and has been found to vary depending on the site [28,29]. Even within the same site, a variation between individuals precludes the use of absolute epithelial thickness alone to indicate pathology. In addition, the assessment of OCT introduces another challenge: large three-dimensional datasets that require interpretation by an expert rater to identify pathologies of interest. Human assessment of each individual frame is intractable, but AI tools are well suited to the rapid interpretation of images. 

We have previously reported a deep learning epithelial segmentation tool [34]. This work leverages that segmentation tool and previously collected endoscopic oral OCT data [30] to develop quantifiable biomarkers that provide direct measurements of oral tissue properties in three dimensions. This work explores the ability of these biomarkers to distinguish lesions from their contralaterals and between disease states (dysplasia through carcinoma). As mild and moderate dysplasia requires no clinical intervention other than monitoring, we also examine the ability of these biomarkers to distinguish dysplastic lesions that later progress to a lesion requiring intervention. And lastly, as this retrospective dataset is inclusive of imaging at multiple time points for a few patients, we explore the reproducibility of measurements and speculate on their potential as a monitoring tool.

## 2. Materials and Methods

### 2.1. Study Design

This is a hypothesis-generating study intended to provide future direction for diagnostic criteria in oral OCT measurement. This work represents a retrospective analysis of a larger imaging trial (n = 123 patients) from 2014–2017 [30]. Imaging was collected of lesions paired with contralateral site measurements. As patients were recruited from a long-term monitoring clinic, some patients were imaged repeatedly over multiple time points. 

**Figure 1:**
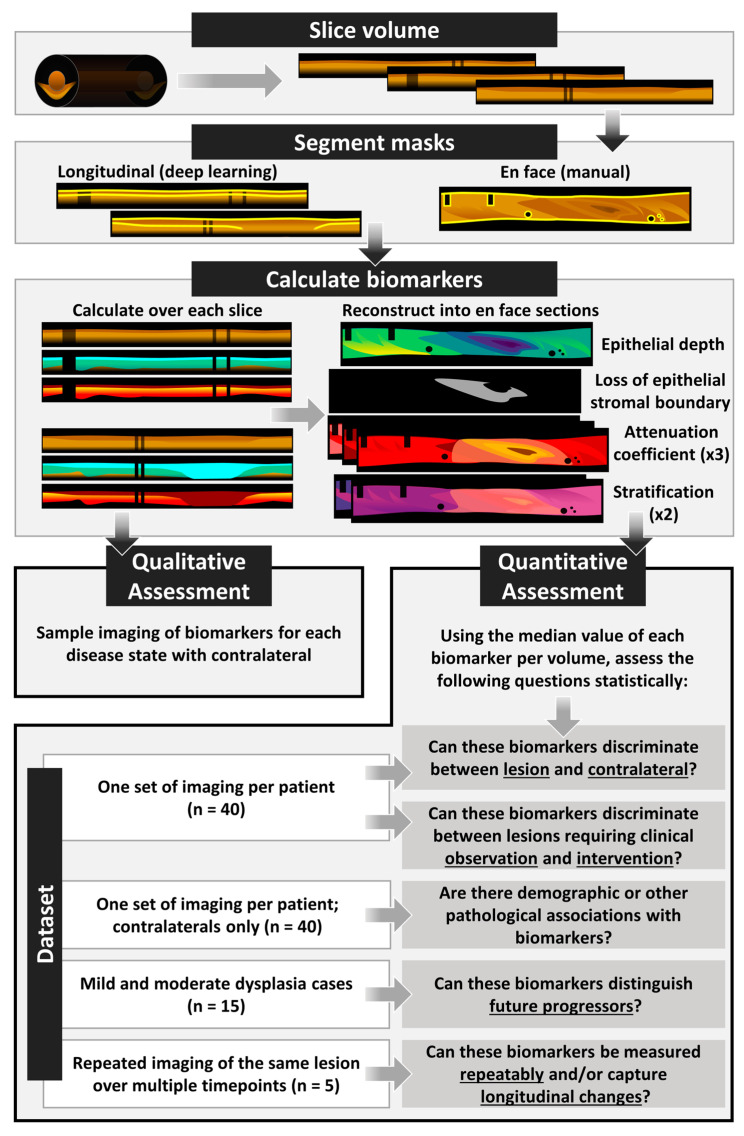
Study design overview.

This work explores the diagnostic utility of seven proposed biomarkers; an overview of the study design and methods can be found in Figure 1. First, we conduct a qualitative analysis of imaging biomarkers across different disease states. Then, we quantitatively assess the following research questions:

- Can the biomarkers discriminate between lesion and contralateral?
- Can the biomarkers discriminate between lesions clinically indicated for observation (benign lesions, mild, or moderate dysplasia) and intervention (severe dysplasia or carcinoma)?
- Are there demographic or other pathological associations with biomarkers?
- Can the biomarkers distinguish future progressors within the mild and moderate dysplasia groups?
- Can the biomarkers be measured repeatably and/or capture longitudinal changes?

Inclusion criteria: For consistency, only images acquired with the same OCT system were selected. While the full dataset includes small numbers of lesions on the lip, soft/hard palate, floor of the mouth, gingiva, or vestibule, most cases are lesions on the tongue. This is as anticipated; the oral tongue is the most common subsite for OSCCs and has the worst prognosis [35,36]. We selected lesions only on the lateral and ventral tongue as they are the most histologically comparable; the dorsal tongue contains papillae that change the appearance of structural features.

Exclusion criteria: Imaging that was of insufficient quality, as characterized by excessive artifacts obscuring the tissue, poor selection of the reference resulting in blurring (assessed by examining the sheath layers), or poor tissue contact (<50% of the volume) with the imaging probe were not included in this study. For all analyses except for the assessment of future progressors, only one time point per patient was used for this study; the selected time point was chosen using a random number generator. 

### 2.2. OCT System

The OCT system used for this study has been described in detail previously [30]. Briefly, a 50 kHz swept-source laser (SSOCT-1310, Axsun Technologies Inc., Billerica, MA, USA) with 20 mW output power feeds a single-mode fiber 90/10 sample/reference split Mach-Zehnder OCT interferometer. The sample arm consists of a fiber-optic rotary joint that connects to a 0.9 mm diameter side-looking rotary-pullback catheter (Dragonfly OPTIS imaging catheter, Abbott Medical Inc., Westford, MA, USA). A custom-built rotary-pullback drive provides two-dimensional scanning at rotational rates up to 100 Hz and pullback lengths up to 90 mm. To minimize artifacts and improve image quality, fiber-based polarization diversity detection was implemented [37].

**Figure 2:**
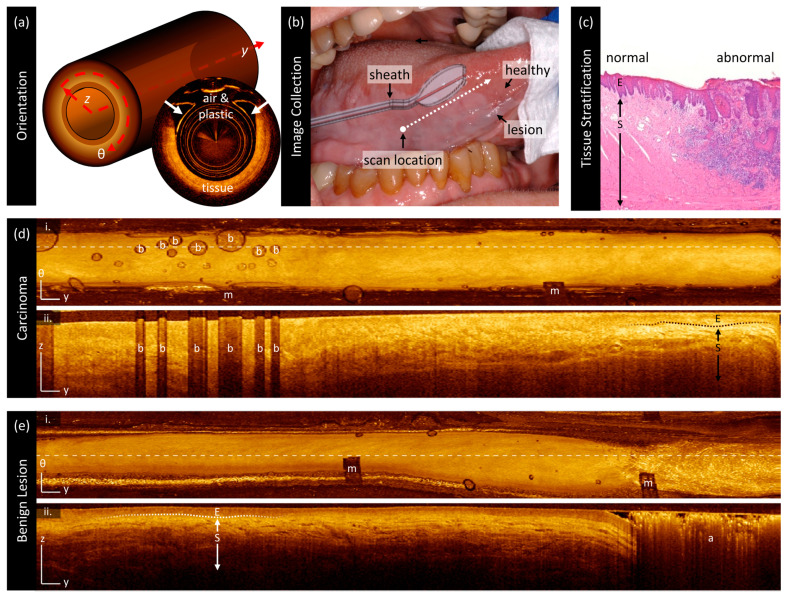
OCT orientation and sample imaging. (**a**) Endoscopic OCT results in a cylindrical volume as shown on the left. When imaging a non-luminal organ, a portion of the imaging field contains no tissue (right). (**b**) The image collection is conducted using a dental mirror or saliva ejector to lay the imaging catheter across the surface of the lesion. (**c**) Histopathology of oral tissue demonstrating epithelial (‘E’) and stromal (‘S’) stratification for comparison against OCT. (**d**) OCT of a squamous cell carcinoma: panel (**i**) demonstrates a mean en face projection and panel (**ii**) demonstrates a longitudinal slice taken from the dashed line shown in (**i**). There are many bubbles (‘b’) present in this volume and sheath markers (‘m’). (**e**) OCT of a benign lesion presented in the same orientation as (**c**). This volume also demonstrates sheath markers (‘m’) and an artifact where tissue is completely obscured (‘a’). In the longitudinal panels (**ii**), the epithelium is the first dark line in the tissue ‘E’ and the stroma is the first bright region ‘S’. The basement membrane itself is not resolvable, but the transition between these two regions (‘epithelial–stromal boundary’) is as denoted by the dashed lines. Scale bars 1 mm.

As shown in Figure 2, this endoscopic imaging system results in long but narrow images, which include a region of non-tissue contact for approximately one-third of the azimuthal direction (Figure 2a). To allow for good access to all parts of the oral cavity, catheters were inserted into a 1.5 mm OD closed-ended plastic sheath that was attached to either a modified saliva ejector with a formable wire or a paddle (Figure 2b) fashioned from a disposable dental mirror with the reflective sticker removed [30]. The sheath was pad printed with black index markers (indicated by ‘m’) spaced every 10 mm to facilitate lesion registration. Whenever possible, index markers were oriented toward the non-tissue side of the catheter holders to not interfere with imaging.

OCT volumes are presented as en face (y-θ) mean intensity projections (Figure 2d,e(i)) and longitudinal (y–z) sections (Figure 2d,e(ii)) where y is the pullback dimension, θ is the circumferential angle around the pullback dimension, and z is the depth into the tissue. The location of longitudinal sections within the en face projections is indicated with dashed lines. The images are oriented with the distal end of the catheter to the left and the proximal end of the catheter to the right. The color scale ranges from black to gold, which we refer to as ‘dark’ or ‘low intensity’ and ‘bright’ or ‘high intensity’ corresponding to the magnitude of light returned from tissue (typically due to backscattering). En face projections are presented with square pixels such that they best represent the geometry of clinical presentation. Longitudinal sections presented in this work are stretched in the A-line direction (z) for better visualization of subtle layering structures. All scale bars are 1 mm.

In longitudinal sections, the epithelium may be visualized as a darkened layer (‘E’) superficial to the brighter stroma (‘S’). An example of how this appears in histopathology is included in Figure 2c for reference. This transition is much more prominent in the benign lesion (Figure 2e) than in the carcinoma (Figure 2d) where there appears to be thicker, brighter epithelium and/or some destruction of the epithelial–stromal boundary such that there is no sharp distinction between epithelium and stroma. Air bubbles (‘b’) in the water between the two catheter sheaths or between the catheter and the tissue appear as oval artifacts in the en face view and as vertical shadowed regions in the longitudinal view. There are other artifacts such as non-uniform rotational distortion (not pictured) or occlusive substances such as mucous or markers present in some images; an example is seen in panel Figure 2e(ii) and is labeled ‘a’. 

### 2.3. Image Collection

This study was approved by the Institutional Review Boards of the British Columbia Cancer Agency and the University of British Columbia (Approval number: H11-02516). Volunteers were recruited from the Vancouver General Hospital Innovative Approach to Triage Oral Precancer (VGH iTOP) clinic and provided informed written consent. The iTOP clinic sees and monitors patients who have premalignant oral lesions.

Patients presenting with oral lesions were examined under white light examination by an oral oncologist (CP or S.N.) followed by autofluorescence evaluation with VELScope. If a biopsy of the lesion was deemed necessary as part of normal clinical care, the following imaging protocol was performed. Clinical impression based on white light and autofluorescence was used to identify the most abnormal part of the lesion that would be biopsied. One of the black markers on the OCT catheter was placed on this site and a volumetric pullback was collected. Imaging on both sides of the black marker was collected straddling the planned biopsy site. Toluidine Blue solution was then applied to the lesion post imaging to assist in the clinical assessment of the lesion. Finally, an incisional or 5 mm punch biopsy was collected and processed following standard practice for histopathological diagnosis. 

### 2.4. Deep Learning Segmentation

This work explores the potential diagnostic utility of quantitative biomarkers. As it is not possible to manually segment each frame of the volumetric OCT data (the dataset described herein represents >35,000 frames), we use a previously developed deep learning pipeline [34] to segment the surfaces of the epithelium and stroma from longitudinal OCT sections. This allows us to calculate biomarkers on each longitudinal slice and generate en face measurements of each volume.

Briefly, this pipeline consists of a series of two classifiers, and two U-Nets were trained on longitudinal sections to (1) identify the field of view (i.e., regions of tissue contact), (2) identify artifacts (bubbles, sheath markers), (3) segment the epithelium surface, and (4) segment the epithelial–stroma interface. Each network was trained and tested independently of each other such that they could be used outside of the pipeline described in [34]. 

The field-of-view network classifies whole longitudinal slices, rejecting those without sufficient tissue contact. The artifact classifiers used a tile-wise approach to generate a rough estimate of regions where the segmentations may not be trustworthy. While this approach is sufficient for visual assessment, quantitative measurement requires more precision in locating the boundaries of tissue contact and removal of artifacts than the tile-wise training approach of the network provided. As such, this work employed the previously described segmentation networks to identify the epithelium region, but the regions of tissue contact and artifacts were segmented manually on mean en face projections by an experienced OCT rater (JM). 

### 2.5. Image Processing

All image processing was conducted in MATLAB 2023a; deep learning predictions were generated using Python 3.6.9 with a PyTorch framework as previously described. All experiments were performed on a Windows 10 operating system, with Intel Core i7-12700K 3.60 GHz CPU, NVIDIA GeForce GTX 3080Ti GPU, and 32 GB of RAM.

**Figure 3:**
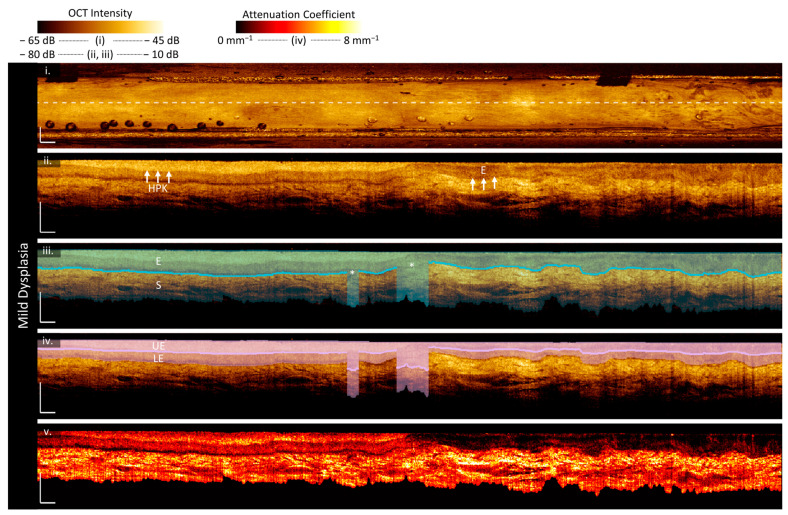
Biomarker calculation on a longitudinal slice. (**i**) OCT mean en face projection. (**ii**) Longitudinal section taken from the dashed line. This sample contains hyperparakeratosis (‘HPK’), which is visualized as a bright layer near the tissue surface on the left-hand side of this scan. The epithelium (‘E’) is darker and present below the hyperparakeratosis on the left. (**iii**) Longitudinal section overlaid with the segmented regions of epithelium (‘E’) and stroma (‘S’). The epithelial–stromal boundary is illustrated by the blue line. There is a region with a loss of epithelial–stromal boundary detection (‘*’) where the entire visualized depth is taken to be epithelium. (**iv**) Longitudinal section overlaid with the intraepithelial regions: upper epithelium (‘UE’) and lower epithelium (‘LE’). (**v**) The depth-resolved attenuation coefficient for this frame. The attenuation coefficient of the epithelium is high in the region containing hyperparakeratosis.

An example of the image processing methods can be seen in Figure 3. First, images are sliced from cylindrical volumes (en face: Figure 3i) into longitudinal frames (Figure 3ii) and rescaled such that each pixel is 10 μm square (using the index of refraction of water as an immersion medium) in both the pullback and A-line direction. The example frame (Figure 3ii–v) has been rescaled in the A-line direction for presentation purposes. Most volumes comprise 504 or 512 longitudinal frames (collected at scan rates of 98 or 100 Hz), 3–9 cm in length, and are collected at a pullback speed of 1–10 mm/s. Rescaling allows for each pixel to represent the same geographic area and is performed with bicubic interpolation. 

Each longitudinal frame was saved as a .tif for interpretation by the deep learning network. After post-processing to remove small gaps as described in [34], the network generated annotation masks containing a single pixel-wide line for the epithelial surface and epithelial–stromal boundary. As shown in Figure 3, the ability of the deep learning network to discriminate between epithelium and stroma is subject to its training data. On the left-hand side of this image (Figure 3ii–v), hyperparakeratosis (‘HPK’) is present as a bright region above the epithelium; however, keratosis was not labeled by raters as part of the training regime for this network, and thus, it is misinterpreted as epithelium. 

The mean en face projection of the volume was segmented using in-house annotation software developed as part of [34]. This segmentation included (1) the region of tissue contact to retain, (2) artifacts to remove (bubbles, sheath markers, other artifacts), and (3) the area of the volume containing the lesion. Lesion annotations were selected using sheath markers for localization against recorded positions and were confirmed against clinical photos. These en face masks are then sliced and scaled in the same fashion as the images to produce a vector containing a flag for each A-line in each longitudinal image.

The epithelial and stromal segmentations are used to define an epithelial region (‘E’) from the surface of the epithelium to the surface of the stroma, and a stromal region (‘S’) from the surface of the stroma to the bottom of the visualized region as shown in (Figure 3iii). The bottom of the visualized region is defined as ending when the signal is 6 dB above the intensity of the noise floor. The noise floor value is calculated from the bottom 25 pixels (250 μm) of the frame. First, A-lines containing no tissue are excluded (i.e., A-lines that contain no epithelial surface segmentation), then the region is smoothed with a 5-pixel Gaussian kernel, and finally, the noise floor is taken to be the mean value of this region. In regions where there is a loss of epithelial–stromal boundary visualization (‘*’) and the surface of the stroma is not identified by the network as shown in the center of the panel (Figure 3iii), the assumption is made that the entire depth of the visualized region is the epithelium. The epithelium and stroma region masks are saved and used for the calculation of biomarkers.

### 2.6. Biomarker Measurement

After pre-processing, each longitudinal frame has a mask for the (1) epithelial region, (2) stromal region, (3) regions to exclude due to poor tissue contact or artifacts, and (4) regions to calculate biomarkers that contain a clinically visible lesion. Seven features that can be calculated on each longitudinal frame are selected for investigation. After calculation, each measurement is reinterpreted as an en face projection as is appropriate for their dimensionality (three-dimensional features are represented as mean en face projections; two- and one-dimensional features do not require additional reinterpretation), allowing the viewer to assess each measurement with the same geometry as their clinical presentation. These measurements are summarized in Table 1: they include morphologic features calculated purely from the epithelial and stromal masks, attenuation features that describe the attenuation coefficient of distinct regions, and stratification features that compare the attenuation coefficient in different regions.

Morphologic features: Epithelial depth is calculated as the height of the epithelial region mask as shown in Figure 3iii, generating one depth measurement per A-line and reported in μm. The loss of epithelial–stromal boundary is a one-dimensional measurement, reported as a percentage of the total region with no stromal surface segmentation compared with the total region of tissue (i.e., containing an epithelial surface segmentation) excluding artifacts. For visualization purposes, this is also saved as an en face mask of regions with a loss of epithelial–stromal boundary visualization that can be displayed in combination with other en face projections. From previous studies in oral OCT, we anticipate that increases in disease status will correspond to an increase in both epithelial depth and loss of epithelial–stromal boundary visualization [17,28]. 

Both morphologic features are subject to challenges associated with the geometry of endoscopic scanning. When the A-line is sampling tissue tangential to the tissue surface for example, the probe begins to lose tissue contact at the limits of the azimuthal scan, the epithelial depth may appear artificially deep and/or the epithelial–stromal boundary may no longer be able to be visualized. This also affects the regions over which attenuation coefficient and stratification features are calculated. We refer to this phenomenon as ‘edge effects’ subsequently.

Attenuation features: The attenuation coefficient (μ) is an optical property of the sample corresponding to the exponential decay of light through the tissue due to scattering and absorption. This provides a quantitative examination of the optical properties of the tissue and is less variable between imaging sessions than intensity, which is subject to fluctuations from the power source, catheter quality, reference position, and user handling. The depth-resolved (3D) attenuation coefficient is calculated using the method described by Liu et al. [38] over a region from the epithelial surface to the bottom of the visualized region (6dB above the noise floor). This produces a new longitudinal section as demonstrated by Figure 3v. Mean en face projections are then taken over the entire depth of visualized tissue (‘overall’), epithelial region, and stromal region. From Yang et al., we anticipate that carcinoma will have a lower overall attenuation coefficient than contralateral though previous work has not examined the epithelium and stroma independently [33].

Stratification features: Last, we propose ratiometric features that compare the attenuation coefficient from different regions. For each A-line, we calculate stratification as a ratio of the difference in attenuation coefficient between two regions over their sum, which produces a normalized value from −1 to +1.

We anticipate epithelial–stromal stratification will capture changes related to the loss of tissue stratification: as the contrast between the layers is low, this value will be near zero and, otherwise, will characterize the direction of attenuation coefficient difference. Second, we explore intraepithelial stratification. Dysplastic grading in oral cancer examines the presence of cellular atypia in each third of the epithelium; however, as healthy epithelium only represents 20–30 pixels (200–300 μm) [29], it was deemed more robust to examine halves rather than thirds. These regions, described as upper epithelium (‘UE’) and lower epithelium (‘LE’), are demonstrated in Figure 3iv.

### 2.7. Quantitative and Statistical Analysis

**Figure 4:**
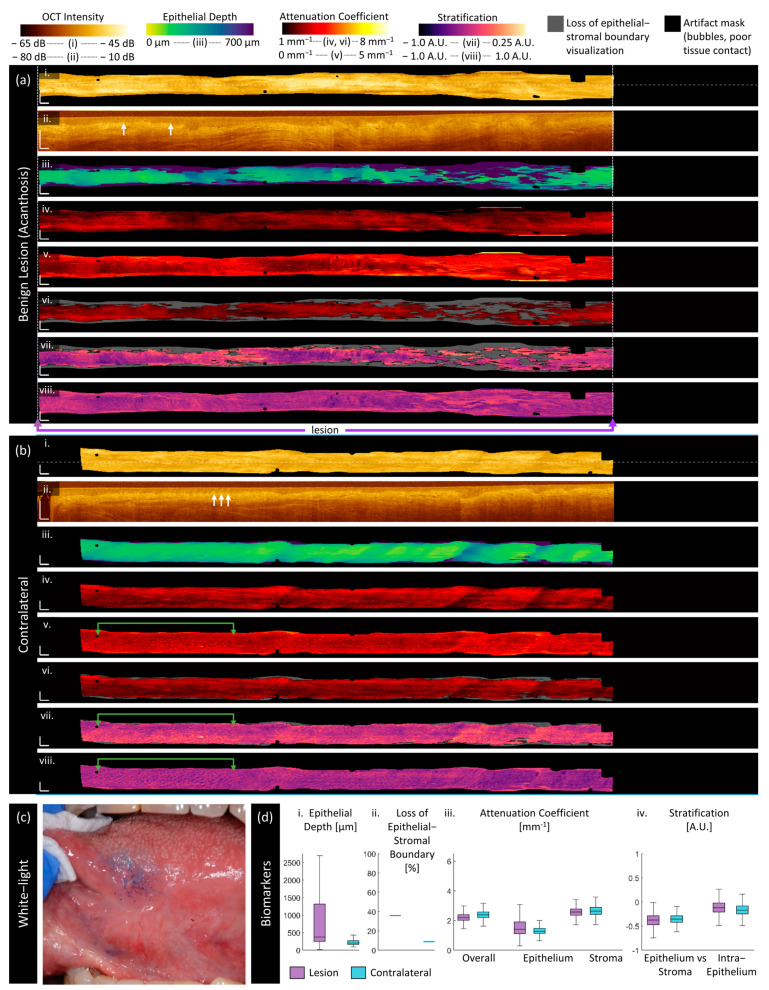
Sample imaging of a benign acanthotic lesion (**a**) and its contralateral (**b**), with the corresponding white light photo (**c**) and box and whisker charts of measurements (**d**). For panels (**a**,**b**): (**i**) OCT mean en face projection; (**ii**) OCT longitudinal slice from the dashed lines in (**i**); (**iii**) epithelial depth; (**iv**) mean projection of overall attenuation coefficient; (**v**) mean projection of epithelium attenuation coefficient; (**vi**) mean projection of stroma attenuation coefficient; (**vii**) epithelial–stromal stratification (**viii**) intraepithelial stratification. Gray regions in panels (**vi**,**viii**) represent a loss of epithelial–stromal boundary visualization. All scale bars 1 mm.

**Figure 5:**
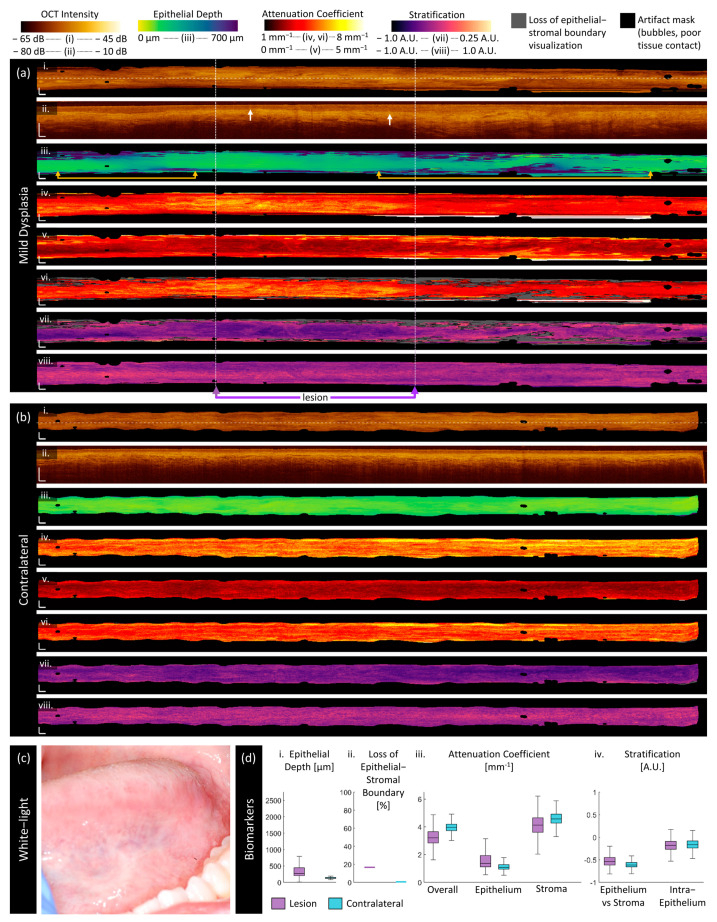
Sample imaging of a mild dysplasia (**a**) and its contralateral (**b**), with the corresponding white light photo (**c**) and box and whisker charts of measurements (**d**). For panels (**a**,**b**): (**i**) OCT mean en face projection; (**ii**) OCT longitudinal slice from the dashed lines in (**i**); (**iii**) epithelial depth; (**iv**) mean projection of overall attenuation coefficient; (**v**) mean projection of epithelium attenuation coefficient; (**vi**) mean projection of stroma attenuation coefficient; (**vii**) epithelial–stromal stratification (**viii**) intraepithelial stratification. Gray regions in panels (**vi**,**viii**) represent a loss of epithelial–stromal boundary visualization. All scale bars 1 mm.

**Figure 6:**
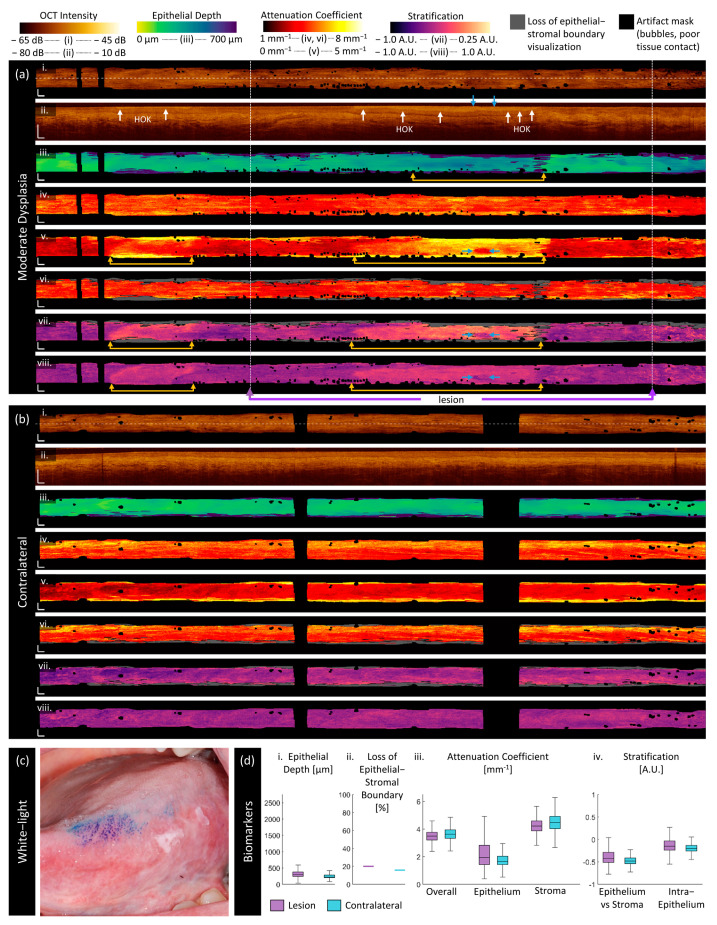
Sample imaging of a moderate dysplasia (**a**) and its contralateral (**b**), with the corresponding white light photo (**c**) and box and whisker charts of measurements (**d**). For panels (**a**,**b**): (**i**) OCT mean en face projection; (**ii**) OCT longitudinal slice from the dashed lines in (**i**); (**iii**) epithelial depth; (**iv**) mean projection of overall attenuation coefficient; (**v**) mean projection of epithelium attenuation coefficient; (**vi**) mean projection of stroma attenuation coefficient; (**vii**) epithelial–stromal stratification (**viii**) intraepithelial stratification. Gray regions in panels (**vi**,**viii**) represent a loss of epithelial–stromal boundary visualization. All scale bars 1 mm.

**Figure 7:**
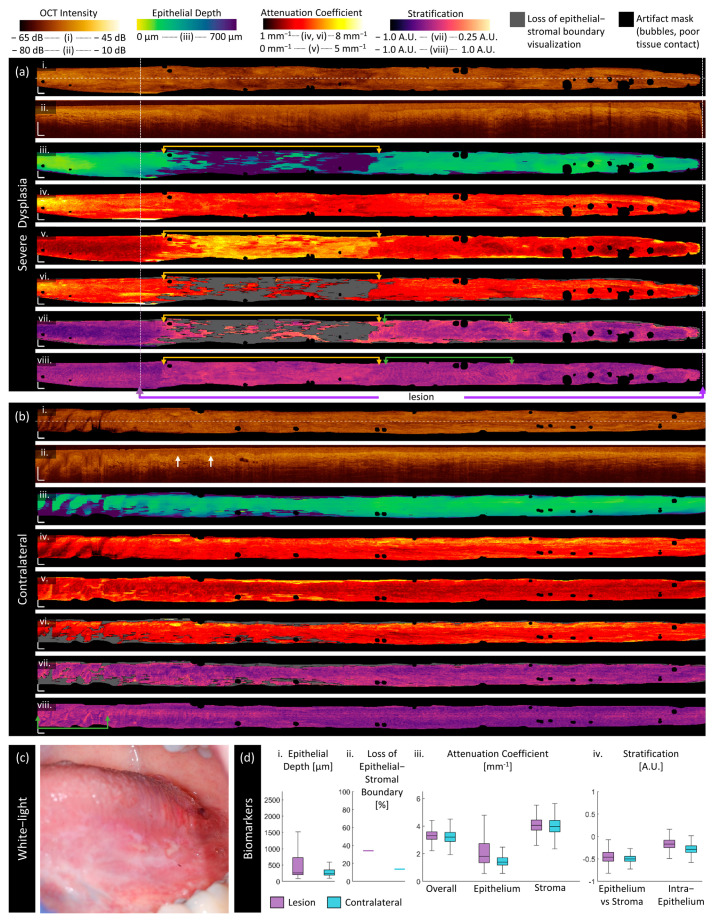
Sample imaging of a severe dysplasia (**a**) and its contralateral (**b**), with the corresponding white light photo (**c**) and box and whisker charts of measurements (**d**). For panels (**a**,**b**): (**i**) OCT mean en face projection; (**ii**) OCT longitudinal slice from the dashed lines in (**i**); (**iii**) epithelial depth; (**iv**) mean projection of overall attenuation coefficient; (**v**) mean projection of epithelium attenuation coefficient; (**vi**) mean projection of stroma attenuation coefficient; (**vii**) epithelial–stromal stratification (**viii**) intraepithelial stratification. Gray regions in panels (**vi**,**viii**) represent a loss of epithelial–stromal boundary visualization. All scale bars 1 mm.

**Figure 8:**
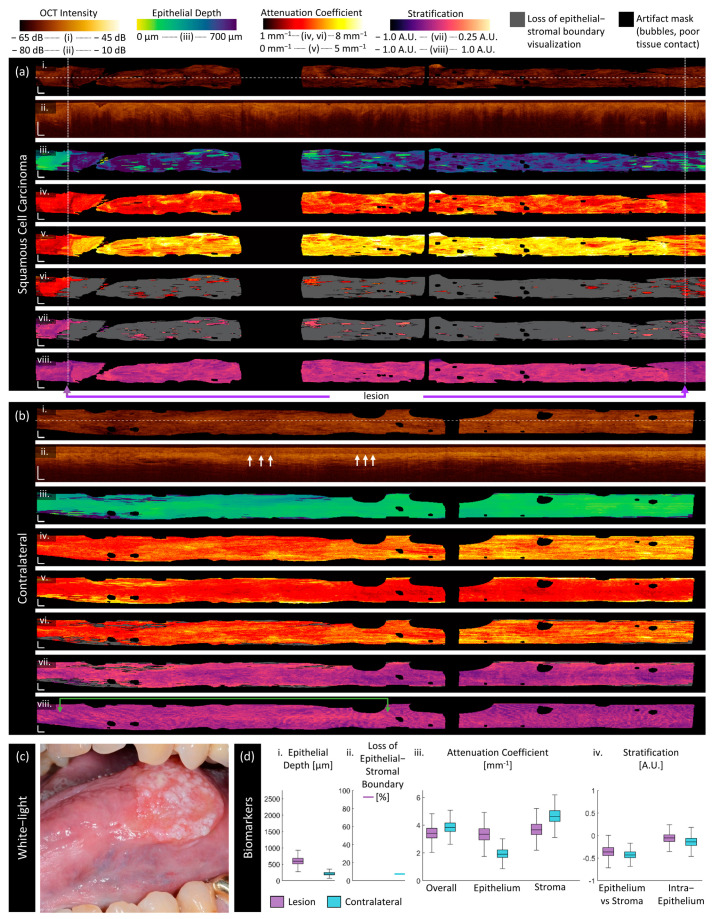
Sample imaging of a squamous cell carcinoma (**a**) and its contralateral (**b**), with the corresponding white light photo (**c**) and box and whisker charts of measurements (**d**). For panels (**a**,**b**): (**i**) OCT mean en face projection; (**ii**) OCT longitudinal slice from the dashed lines in (**i**); (**iii**) epithelial depth; (**iv**) mean projection of overall attenuation coefficient; (**v**) mean projection of epithelium attenuation coefficient; (**vi**) mean projection of stroma attenuation coefficient; (**vii**) epithelial–stromal stratification (**viii**) intraepithelial stratification. Gray regions in panels (**vi**,**viii**) represent a loss of epithelial–stromal boundary visualization. All scale bars 1 mm.

For most biomarkers (except for the one-dimensional loss of epithelial–stromal boundary visualization), each volume includes a measurement for every A-line. This is demonstrated through box and whisker plots in Figure 4, Figure 5, Figure 6, Figure 7 and Figure 8 (box: upper and lower quartiles; bar: median; whiskers: maximum and minimum). These plots summarize the quantitative measurements for all pixels in the area labeled ‘lesion’ in the volume and all pixels in the contralateral volume. This represents thousands of data points, some of which may include tissue outside of the area of the lesion or regions with edge effects. 

For statistical analysis, a single median value is used for each biomarker of each volume. Only data points within areas of tissue contact are included in the calculation of the median value; all regions labeled as artifacts are excluded. To minimize the impact of edge effects on quantitative analysis, a vertical (azimuthal) erosion operator is applied to the en face tissue contact mask such that only the central 50% of the tissue mask is retained. For lesions, only data points within the labelled lesion area are included. Statistical analysis is conducted in TIBCO Statistica 14.

This is a hypothesis-generating study intended to provide future direction for diagnostic criteria in oral OCT measurements. Our sample size is relatively small, and though it is sufficient to allow for examination of subgroups (e.g., disease states, sex, and age), we cannot control for confounding covariates. As larger scale studies will be required to confirm any findings described in this paper, we generally prioritize reducing type II errors. We have selected a significance level of *p* < 0.05 for all tests and present *p* values without correction for multiple comparisons.

The Shapiro–Wilk W test [39] is used to test biomarkers for normality and assess which features require parametric or non-parametric tests. For paired data (lesion/contralateral, male/female), Welch’s paired *t*-test [40,41] is used for parametric features, and the Wilcoxon rank-sum [42] for non-parametric features. Missing data are excluded in a pairwise fashion. To assess associations between the proposed metrics and diagnosis (clinically indicated for observation/intervention), the unpaired *t*-test [43] was used for parametric features and the Mann–Whitney U test [44] for non-parametric features. Homogeneity of variances was assessed with Levene’s test [45], and the presence of outliers was assessed with Grubb’s test [46]. 

To reduce the effect of disease state, statistical analysis of demographic features and other pathologic conditions (acanthosis, keratosis, mucositis, smoking status, age, and sex) was only performed on contralaterals. For the assessment of biomarkers against sex, smoking status, and other biological conditions, the unpaired *t*-test was used for parametric features and the Mann–Whitney U test for non-parametric features. To assess associations between measurements and age, Spearman’s rank order [47] was used.

For future progression, we are only concerned with changes to the area of the lesion. As such, we normalize each lesion to its respective contralateral by subtracting the median contralateral measurement from the median lesion measurement. Cases without a matched lesion and contralateral were excluded. Again, the unpaired *t*-test was used for parametric features, and the Mann–Whitney U test for non-parametric features.

Last, no statistical analysis was conducted on the longitudinal assessment of biomarker repeatability as there was no repetition of patients with the same disease state over multiple time points that met the inclusion criteria. Only qualitative assessments of trends are discussed in this experiment.

## 3. Results

### 3.1. Datasets and Demographics

Table 2 describes the demographic breakdown of the patients in this study to assess whether the proposed biomarkers can discriminate between lesion and contralateral and/or between disease states. 

To allow for sufficient sample size for each group, all carcinomas are described as one group; though there is one verrucous carcinoma, all others are squamous cell carcinomas. We use ‘benign’ to describe lesions that are histopathologically confirmed as non-cancerous and non-dysplastic. The five benign cases are the following:

- Oral candidiasis with focal ulceration and intense chronic mucositis;
- Lichen mucositis, hyperorthokeratosis;
- Lichenoid mucositis with marked hyperorthokeratosis;
- Mild acanthosis, basilar proliferation, no dysplasia but history of SCC at this site;
- Acanthosis.

Contralateral images were taken from the side of the tongue opposite the lesion whenever possible. However, as the patients in this study were recruited into a long-term monitoring group due to a history of or risk of oral cancers, we cannot assume the contralateral samples represent healthy normal tissue, only that they contained no clinically visible lesions.

The mean age of patients recruited in this study was 58 (29–92). Seventeen patients were imaged with lesions on the lateral tongue and twenty-three with lesions on the ventral tongue. The benign lesions exhibited lichenoid mucositis (n = 3) and hyperorthokeratosis (n = 2). Hyperkeratosis was present in 19% of dysplastic cases, and hyperparakeratosis was present in 35%. Eighteen of these patients did not smoke (‘never smokers’), seven were former smokers, and five were current smokers (twelve ‘ever smokers’) at the time of imaging.

In some cases, contralateral and lesion data are not perfectly paired. Forty patients are included in this study (twenty males and twenty females). There is one case included of a contralateral with no corresponding lesion site imaged and nine lesions with no corresponding contralateral. A plurality of the lesions with no corresponding contralateral are carcinoma cases (n = 4/9) as these were patients imaged at the time of surgical intervention.

All mild and moderate dysplasias with contralaterals were included for assessment of future progression. Clinical status from the time of imaging (July 2014–June 2017) to the time of writing (April 2024) was reviewed. Patients diagnosed with severe dysplasia, or carcinoma during the follow-up time were considered ‘progressors’; patients without a lesion of a higher disease status were considered ‘non-progressors’ (Table 3). The average time from imaging to diagnosis for progressors was 45 months (22–60 months). At the time of imaging, all progressors (n = 4) presented with acanthosis and hyperparakeratosis or hyperkeratosis. This was less consistently present in the non-progressors (n = 14): acanthosis (6/14), hyperparakeratosis (4/14), hyperkeratosis (3/14), and hyperorthokeratosis (1/14). 

To assess whether the proposed biomarkers can be measured repeatably and/or capture longitudinal changes, five patients (four male and one female) with lesions imaged at multiple time points met the inclusion criteria. They are described in Table 4. This represented one patient for each diagnostic state. No patients underwent any surgical or clinical intervention between imaging time points. Patients 1 and 3 presented with hyperparakeratosis and acanthosis; Patient 4 presented with hyperkeratosis. 

### 3.2. Sample Imaging

**Figure 9:**
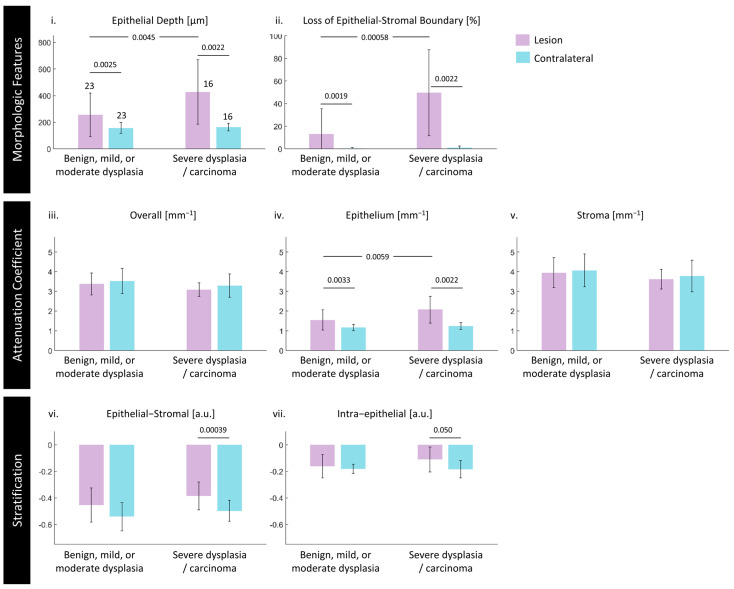
Comparison of lesions clinically indicated for observation (benign lesions, mild, or moderate dysplasia) and intervention (severe dysplasia or carcinoma) against their contralaterals: (**i**) epithelial depth, (**ii**) loss of epithelial–stromal boundary visualization, (**iii**) overall attenuation coefficient, (**iv**) epithelium attenuation coefficient, (**v**) stroma attenuation coefficient, (**vi**) epithelial–stromal stratification, (**vii**) intraepithelial stratification. Lesions are reported in purple bars and contralaterals in blue. The height of the bars is the mean value of medians of all volumes in that category; the error bars are the standard deviation of the medians. The sample size for each group is presented over the bars in (**i**). Statistically significant differences (*p*-values) are indicated as bars above compared groups; all other comparisons were found to be not significant (*p* > 0.05).

We present sample imaging of each disease state reported in this work in Figure 4, Figure 5, Figure 6, Figure 7, Figure 8 and Figure 9. Each figure contains the measurements of the lesion (a) and contralateral (b) as well as the clinical view of the lesion (c) and box and whisker plots of the quantitative measurements of each biomarker (d). All scale bars are 1 mm, and all figures are presented with the same colormap scaling for each feature.

Benign lesion:

Figure 4 is imaging of an 81-year-old male patient with biopsy-confirmed acanthosis with no dysplasia on the left ventral tongue. A photograph of the lesion after application of toluidine blue is included in Figure 4c. The area of the lesion as determined clinically is the entire length of the scan (purple arrows below panel a); this area is used for the lesion measurement in the box and whisker plots (panel d). 

The longitudinal scan (Figure 4a(ii)) demonstrates the hallmarks of acanthosis: thickened and irregular epithelium. This is captured in the en face epithelial depth measurement, which is higher in the lesion (Figure 4a(iii)) than its contralateral (Figure 4b(iii)), with regions of extreme epithelial depth corresponding to a loss of epithelial–stromal boundary visualization, which appears as gray regions in Figure 4a(vi,vii) panels. The loss of the epithelial–stromal boundary in the contralateral is only found at the edges of the volume and, thus, is likely due to ‘edge effects’. However, the epithelium in the contralateral also appears abnormal with some of the same features as the lesion: ripple-like changes in depth along the length of the scan and small papillae extending from the epithelial–stromal boundary (white arrows) (Figure 4a,b(ii)). 

The attenuation coefficient measurements are lower than their contralateral counterparts and, also, lower than the dysplastic lesions presented in Figure 5a and Figure 6a. Regions of higher epithelial depth correspond to regions of decreased overall attenuation coefficient (Figure 4a(iv)) and increased epithelial attenuation coefficient (Figure 4a(v)). The stroma attenuation coefficient (Figure 4a(vi)) appears similar to the overall attenuation coefficient (Figure 4a(iv)).

The epithelium attenuation coefficient (Figure 4b(v)) captures small pockmarks, which are further emphasized in the intraepithelial stratification (Figure 4b(vii)), highlighted by the green arrows. These appear as small bright dots in the epithelium attenuation coefficient (Figure 4b(v)) and dark dots in the intraepithelial contrast (Figure 4b(viii)), indicating that they are changes derived from the lower epithelium. From the longitudinal scan (Figure 4b(ii)), this appears to map to the papillae (white arrows) extending from the epithelial–stromal boundary. These features are more apparent in the contralateral and left side of the lesion where the epithelium is thinner and flatter; they are overshadowed by larger spatial changes which correspond to areas of a loss of epithelial–stromal boundary visualization.

Mild dysplasia:

Figure 5 is imaging of a 47-year-old male patient with mild dysplasia, hyperparakeratosis, and acanthosis on the left ventral tongue as photographed in panel (c). The boxed region in the center of the scan (purple arrows) shows where the clinically identified margins of the lesion were identified and is the region that the lesion box and whisker plots (panel (d)) are calculated over. However, examination of the proposed biomarkers indicates this may not include all the abnormal tissue and may not include the most pathologic regions. Two abnormal regions of epithelial depth (Figure 5a(iii)) are indicated with gold arrows and are outside the clinically selected region.

In the longitudinal OCT sections, the lesion volume (Figure 5a(iii)) has a deeper and more irregular epithelium than its contralateral (Figure 5b(iii)). The contralateral is extremely regular, with very few changes evident in the epithelial depth map. Examining the measurements in Figure 5d(i), the median lesion epithelial depth measurement is nearly double the contralateral and the depth varies more over the volume. There are several regions of extreme epithelial depth visible in the en face epithelial depth map (Figure 5a(iii)), which correspond to a loss of epithelial–stromal boundary visualization. Some of these regions may be artificially high due to edge effects, which appear to be more prominent in the lesion (perhaps due to surface texture impacting catheter contact) but are still present in the contralateral.

Areas with thicker epithelium have a lower overall attenuation coefficient (Figure 5(iv)), higher epithelium attenuation coefficient (Figure 5a(v)), and lower stroma attenuation coefficient (Figure 5a(vi)) where the stroma is still visualized. All attenuation coefficients are higher in this patient than in the patient with the benign lesion (Figure 5a). The overall and stroma attenuation coefficients are lower in the lesion than in the contralateral, but this relation is reversed in the epithelium. 

The epithelial–stromal stratification (Figure 5a(vii)) is increased in the region with greater epithelial depth. There are also textural features in the epithelium attenuation coefficient of the lesion (Figure 5a(v)), which are emphasized in the epithelial–stromal stratification. These textural features are not present in the contralateral, which is regular throughout the volume. 

The intraepithelial stratification is consistent across the length of the lesion (Figure 5a(viii)) and contralateral (Figure 5b(viii)). There are a few bright papillae extending from the epithelial–stromal boundary through the epithelium (white arrows, Figure 5a(ii)), but fewer than in the benign lesion and the pockmark pattern is not as prominent in either stratification metric (Figure 5a(vii,viii)).

Moderate dysplasia:

Figure 6 is imaging of a 67-year-old male patient with moderate dysplasia, hyperorthokeratosis, and acanthosis on the left ventral tongue. The photograph in panel (c) shows the lesion after application of toluidine blue. As in the mild dysplastic case (Figure 6), the clinically selected area of the lesion (purple arrows) does not encompass all the abnormal-appearing regions (gold arrows, Figure 6a). The measurements of lesion presented in panel (d) are from the region indicated by the purple arrows, and are inclusive of both abnormal-appearing tissue (gold arrows) and less abnormal tissue. The hyperorthokeratosis (‘HOK’, white arrows) is visible in the longitudinal section (Figure 6a(ii)). 

The epithelial depth measurements are qualitatively (Figure 6a(iii)) and quantitatively (Figure 6d(i)) similar to the mild dysplasia sample (Figure 5), with increased epithelial depth across the volume compared with their contralaterals and small regions of distinctly increased epithelial depth that correspond to the loss of epithelial–stromal boundary visualization. In this patient, the contralateral epithelial depth (Figure 6b(iii)) is higher and contains more ‘edge effects’ than in the mild dysplasia case.

The epithelium attenuation coefficient (Figure 6a(v)) reveals potential margins that are not visible in the en face OCT (gold arrows). These regions have a much higher epithelium attenuation coefficient than the surrounding tissue or the contralateral. However, they also correspond with the regions of hyperorthokeratosis (‘HOK’) visible in the longitudinal section (Figure 6a(ii)). Hyperorthokeratosis will be included in the epithelial region mask, driving the measured attenuation coefficient up. This effect is especially visible in the region indicated by blue arrows where there is no hyperorthokeratosis, which appears as a dark circle compared with its surroundings in the epithelial attenuation coefficient and both stratification features.

The stratification features provide a better visual discrimination of the regions indicated by gold arrows although the margins differ slightly in Figure 6a(vii,viii). From the epithelial–stromal stratification (Figure 6a(vii)), we see that there is an increase in the relative epithelial attenuation coefficient. From the intraepithelial stratification (Figure 6a(viii)), it is clear that this change is driven by an increase in the upper epithelium, which corresponds to the region we expect the hyperorthokeratosis to be present in. There are also some textural changes visible at the distal and proximal boundaries of the lesion volume. 

There is an increase in epithelial depth (Figure 6a(iii)) at the right-hand side of the image, which does not have corresponding increases in epithelial attenuation coefficient or stratification measurements. This could be either due to an ‘edge effect’ at the end of the OCT acquisition, which could occur due to catheter positioning or movement. However, upon examining the longitudinal section, it appears that this region does not present with the same hyperorthokeratosis, and this region may be an accurate representation of epithelial changes.

Severe dysplasia:

Figure 7 is OCT collected of a 56-year-old male patient with severe dysplasia, hyperparakeratosis, and acanthosis on the left ventral tongue. A photograph of the lesion is included in panel (c). In this case, the clinically selected area of the lesion (purple arrows, measurements in panel (d)) encompasses the area of abnormality as identified by the proposed biomarkers (gold arrows, Figure 7a) but is less specific to the most abnormal region. Many of the features present similarly to the mild dysplastic case (Figure 7a): deep epithelium, high attenuation coefficient, loss of epithelial–stromal boundary, and higher stratification in both the epithelial–stromal and intraepithelial biomarkers compared with their respective contralaterals. This case does not present with keratosis visible in the longitudinal scan (Figure 7a(ii)) or throughout the lesion in the same manner as Figure 6a. 

An examination of the longitudinal scan (Figure 7a(ii)) shows that the epithelial–stromal boundary is indistinct in the region indicated by gold arrows. This is largely unsegmented by the deep learning network, resulting in most of the area being classified as the loss of epithelial–stromal boundary. An examination of the margins of the lesion shows epithelial depth increasing as it approaches the area with the loss of epithelial–stromal boundary visualization. 

The epithelial–stromal stratification (Figure 7a(vii)) is darker and smoother on the left side of the volume, which is outside of the clinical boundaries for the lesion. On the right-hand side of the lesion, there are ripples and whorls (green arrows, Figure 7a(vii)), which appear as intensity changes and waves in the epithelial–stromal boundary in the longitudinal section (Figure 7a(ii))). These features are emphasized in the intraepithelial stratification (green arrows, Figure 7a(viii)) where smaller ripples appear more prominently. This is especially visible on the left-hand side of the contralateral (green arrows, Figure 7b(viii)) where there is substantial distortion of the epithelial–stromal boundary, papillae extending into the epithelium (white arrows), and bulb-shaped epithelial protrusions into the stroma. However, some sharp discontinuities in the imaging suggest there may be motion artifacts adding to this effect.

Carcinoma:

Figure 8 is imaging from a 76-year-old female patient with squamous cell carcinoma on the right ventral tongue as photographed in panel (c). The clinically selected area of the lesion (purple arrows) encompasses nearly the entire volume, which corresponds well with the areas of abnormality visualized by the biomarkers. The box and whisker plots (d) indicate 

As visible in the longitudinal section (Figure 8a(ii)), there is no clear epithelial–stromal boundary throughout most of this volume. Therefore, the epithelial depth (Figure 8a(iii)) appears very deep, and no measurement can be made for most of the stroma attenuation coefficient (Figure 8a(vi)) and epithelial–stromal stratification (Figure 8a(vii)). The epithelial attenuation coefficient is high (Figure 8a(v)) as is the intraepithelial stratification (Figure 8a(viii)), indicating that the attenuation is increased at the surface. 

In the contralateral (Figure 8b(ii)), we again see papillae extending from the epithelial–stromal boundary into the epithelium along the length of the volume (white arrows), though they appear more as dark bands rather than the bright (high scattering) features previously demonstrated. This leads to a ripple-like pattern (green arrows) in the intraepithelial stratification (Figure 8b(viii)) with wider peaks and troughs than the ripples in the severe dysplasia case.

### 3.3. Quantitative Assessment of Disease Status and Contralaterals

We present measurements of each feature for all volumes against the disease status in Figure 9. Samples are grouped into lesions clinically indicated for observation (benign lesions, mild, or moderate dysplasia) and intervention (severe dysplasia or carcinoma). A detailed breakdown per diagnosis is available in Supplementary Table S1. All quantitative measurements are calculated after masking out the edge effects as demonstrated in Section 3.2 and assessed for statistical significance via the methods discussed in Section 2.7. Using the Shapiro–Wilk W test, epithelium depth, loss of the epithelial–stromal boundary, epithelium attenuation coefficient, and intraepithelial stratification were found to not have normal distributions and were treated as non-parametric features for this analysis.

Lesion vs. Contralateral: Using the paired statistical tests described in Section 2.7, significant differences (*p* < 0.05) were found between the lesion and the contralateral for both groups in epithelial depth, loss of epithelial–stromal boundary visualization, and the epithelial attenuation coefficient, and just in the severe dysplasia/carcinoma group, for the two stratification metrics. 

Morphologic features: The contralateral measurements are consistent across all disease states: the median epithelial depth (Figure 9(i)) of contralateral volumes is 160 μm and the mean loss of epithelial–stromal boundary visualization (Figure 9(ii)) is marginal (1%). Both features have a statistical difference between lesions requiring observation and intervention. Loss of epithelial–stromal boundary visualization appears to provide the best discrimination—in particular, carcinoma (mean 77%; see Supplementary Table S1) appears entirely distinct from benign lesions (8%), mild (5%) or severe (15%) dysplasia. Epithelial depth follows a similar trend, although there is less separation between mean values.

Attenuation coefficient features: These features have more overlap when compared with the morphologic features and less consistency in the contralateral measurements across disease states in the overall and stroma attenuation coefficients. The median attenuation coefficient for all contralaterals is 3.45 mm^−1^ overall (Figure 9(iii)); 1.20 mm^−1^ epithelium (Figure 9(iv)); 3.97 mm^−1^ stroma (Figure 9(v)). While the epithelial attenuation coefficient tends to be lower than the stroma, it increases with disease status whereas the stroma attenuation coefficient decreases. 

The epithelium attenuation coefficient displays the similar discriminatory ability to the morphologic features, with significant differences between lesions requiring intervention and observation, as well as significant differences between each group and their respective contralaterals. The stroma and overall attenuation coefficients display no significant differences; the overall attenuation coefficient appears to be dominated by the stromal contribution.

Stratification features: The epithelial–stromal stratification (Figure 9(vi)) is consistently negative as the stroma attenuation coefficient is consistently greater than that of the epithelium, but the differentiation between the epithelium and stroma is lower in lesions requiring intervention. For both groups, the contralateral is lower than the lesion, representing a stronger contrast between the epithelium and the stroma as anticipated. There is a significant difference between lesions requiring intervention compared with their contralateral.

The intraepithelial stratification (Figure 9(vii)) reveals subtler differences but follows the same trends. The intraepithelial stratification is again consistently negative, indicating that the epithelial attenuation is higher in the region closest to the epithelial–stromal boundary. Contralaterals are lower in value (more stratified) than their respective groups; there is a significant difference between lesions requiring intervention and their contralaterals.

### 3.4. Demographic and Other Pathologic Associations

A complete table presenting these biomarkers divided by disease state and per sex is available in the Supplementary Materials in Table S2. There is a significant (*p* < 0.05) difference between male (mean 180 µm) and female (mean 140 µm) epithelial depth.

There is a positive correlation (ρ = 0.47, *p* < 0.001) between age and epithelium attenuation coefficient. Breaking this down per sex, there are no significant correlations between age and any biomarker for males, but there are two positive correlations between age and epithelium attenuation coefficient (ρ = 0.52, *p* < 0.05) and age and epithelial–stromal stratification (ρ = 0.59, *p* < 0.05) for females.

Due to sample size limitations (n = 2 lichenoid mucositis, n = 2 hyperorthokeratosis), we were limited in our ability to assess the other pathologic conditions described in Section 3.1. No statistical difference was found in contralaterals with or without acanthosis, or between contralaterals with or without keratosis (inclusive of hyperkeratosis, hyperorthokeratosis, or hyperparakeratosis). Detailed results are presented in the Supplementary Materials in Table S3.

We assessed the contralateral measurements of all biomarkers with respect to smoking status. Ever smokers demonstrate a significantly lower epithelial stratification (*p* = 0.015) in their contralateral measurement: there is a greater stratification between the upper and lower epithelium attenuation coefficients in these patients. There were no other significant differences between ever smokers and never smokers or between current and former smokers.

### 3.5. Future Progression

**Figure 10:**
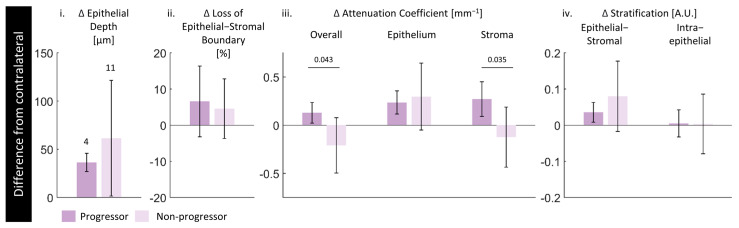
Measurements against future progression for mild and moderate dysplasias: (**i**) epithelial depth, (**ii**) loss of epithelial–stromal boundary visualization, (**iii**) attenuation coefficients, (**iv**) stratification. Future progressors are reported using dark purple bars and non-progressors using light purple bars. The height of bars is the mean value of medians of all volumes after subtracting the median of their contralateral; the error bars are the standard deviation of the same. The sample size for each group is presented over the bars in (**i**). Statistically significant differences (*p*-values) are indicated as bars above compared groups; all other comparisons were found to be not significant (*p* > 0.05).

To examine whether the proposed biomarkers can distinguish future progressors within the mild and moderate dysplasia groups, we present the measurements of each volume normalized to their respective contralaterals in Figure 10. Further details are presented in the Supplementary Materials in Table S4.

There are more non-progressors than progressors, and the standard deviation in non-progressors is higher in all measurements. The best separation is in the stroma and overall attenuation coefficients (Figure 10iii) where the progressors are higher than their respective contralaterals compared with the non-progressors, which are lower than their respective contralaterals. In both lesion and contralateral, these are higher in the non-progressors for overall (3.45/3.32 mm^−1^ lesion/contralateral progressors to 3.60/3.81 mm^−1^ non-progressors) and stroma attenuation coefficient (4.04/3.77 mm^−1^ progressors to 4.35/4.47 mm^−1^ non-progressors). 

In general, progressors present with a lower (though not significant) difference in epithelial depth compared with their contralaterals (Figure 10i), more loss of epithelial–stromal boundary (Figure 10ii), and less change in epithelial–stromal stratification (Figure 10iv). The epithelium attenuation coefficient and intraepithelial stratification are both very similar between progressors and non-progressors (Figure 10iii).

### 3.6. Reproducibility and Repeatability

Next, we explore whether the proposed biomarkers can be measured repeatably and/or capture longitudinal changes in Table 5 with the five patients who were imaged at multiple time points. We present contralateral measurements over the two time points to assess long-term reproducibility. We present repeated measurements (single time point) of lesions to assess short-term repeatability.

All five patients had contralaterals imaged at both time points. For almost all cases, the mean contralateral measurements are within the ranges presented in Figure 9 and Tables S1 and S2. The average difference normalized to the mean measurement of each feature for all five patients is as follows: 20% epithelial depth; 30% loss of epithelial–stromal boundary visualization; 15% attenuation coefficient; 10% epithelium attenuation coefficient; 20% stroma attenuation coefficient; 6% epithelial–stromal stratification; 27% intraepithelial stratification. This indicates that the morphologic masks are the least repeatable between time points. This is somewhat expected: as several months passed between imaging time points, the contralateral site imaged may not be at the same location. 

Two patients had lesions that were imaged repeatedly at a single time point: mild dysplasia (Patient 1) and moderate dysplasia (Patient 2). These demonstrate good repeatability of measurement within a single time point: they represent taking an image, removing, and readjusting the imaging catheter on the lesion, and taking another image. The average difference normalized to the mean measurement of each feature for these patients is as follows: 16% epithelial depth; 77% loss of epithelial–stromal boundary visualization; 3% attenuation coefficient; 20% epithelium attenuation coefficient; 1% stroma attenuation coefficient; 14% epithelial–stromal stratification; 11% intraepithelial stratification. These results are slightly more consistent than the longitudinal contralateral experiment, but there is still some variation in measurements, which may be due to heterogeneity of the lesion and positioning of the imaging catheter.

## 4. Discussion

### 4.1. Dataset Limitations

This work is a retrospective analysis, and as such, there are some limitations in the dataset size, composition, and image collection approach. To improve the sample size, we have grouped together site and pathologic groups, which may merit an independent study. This work combines imaging of the lateral (n = 16) and ventral (n = 23) tongue. Additionally, we have combined mild and moderate dysplasias with benign lesions covering a range of pathologies (‘observation’) as well as severe dysplasia and carcinoma (‘intervention’). 

Our findings are limited to the co-registered labels of the lesion area within each volume. The identified clinical margins are reliant on the 1 cm sheath markers and estimation of lesion size and may not be precisely co-registered. Some of the sample imaging, such as Figure 5, indicate that the selected margins may be wider than (or may not overlap with) areas of abnormality as visualized by the proposed measurements. As we selected the median value of each measurement over the clinically selected margins as our primary quantitative metric, the inclusion of areas of less- or non-pathologic tissue may have reduced differences between the lesion and the contralateral. Additionally, due to the heterogeneous nature of oral cancer, we may have imaged a region that included higher- or lower-grade lesions than the biopsy results, which may have resulted in some deviation between the disease states.

### 4.2. Morphologic Measurements

Epithelial depth and changes in the visualization of stratification have previously been described in the oral OCT literature [17,28,31]. The increase in epithelial depth and irregular epithelial stratification is a well-documented histopathologic hallmark of dysplastic progression to carcinoma [4]. This work represents the first measurement of these features in an endoscopic OCT system. Previously, the healthy ventral tongue is reported to be measured with OCT as 240 µm (160–320 µm) for 28 healthy volunteers with a mean age of 36 years [29], which is higher but comparable to our findings of 160 µm (120–260 µm) of contralaterals from patients undergoing monitoring for oral lesions with a mean age of 58 years. 

Our measurements are limited by the training approach taken in the previously developed deep learning segmentation pipeline [34]. A substantial limitation is poor labeling of keratosis: as these regions are labeled as epithelium, this results in increased epithelium depth as well as subsequent changes in the attenuation coefficient and stratification measurements. Additionally, we have rescaled pixels assuming all values are traveling through a medium with the index of refraction of water: this assumption may not hold in all cases. If the index of refraction is over- or underestimated, the rescaled pixels will no longer be 10 µm, which will skew the measurement of the epithelial depth.

A major caveat of this work is our methods for regions where the stroma surface is not segmented. In our approach, regions with a loss of epithelial–stromal boundary visualization are labeled such that the epithelial mask contains the entire depth of visualized tissue. The underlying assumption we are making is that the epithelial–stromal boundary is now beyond the viewing range of our system. Previous work has shown that cancer-involved margins have a mean epithelial depth on the order of 580 µm (130–900 µm) [20], which would often be beyond our maximum visualized depth (~750 µm in carcinoma, though it varies with attenuation coefficient)—and even if it is within the visualized depth, the contrast lessens deeper into the A-line, which may limit the ability to distinguish tissue layers. 

The result of this assumption is higher than usual epithelium depth measurement in regions with a loss of a visualized epithelial–stromal boundary. This also impacts other features as well: the epithelium attenuation coefficient will be taken over a larger region, and the intraepithelial stratification may not represent the ‘upper’ and ‘lower’ regions well. However, using epithelium measurements only over regions of good epithelial–stromal boundary visualization substantially limits the utility of the proposed technique: excluding regions with a loss of epithelial–stromal boundary will exclude the most pathologic regions in cases of the disease. In sum, we recommend the reader interpret the measurements presented in this work carefully, particularly those in carcinoma where there is a high loss of epithelial–stromal boundary visualization.

### 4.3. Attenuation Coefficient Measurements

In this study, we are unable to retrospectively characterize and compensate for the confocal effects of each optical catheter so we caution that our attenuation coefficient results may not be generalizable to other applications. However, as lesions and contralaterals were imaged at the same time, we are confident that our findings are not obscured by differences between catheters—there were only four unique catheters used to collect the data presented in this study. Previous work in our group has found that the effects are minimal in regions with attenuations of 2–3 mm^−1^ throughout our imaging range; however, findings in this study for the stroma attenuation coefficient are beyond that range and may be unreliable. In addition, given that each imaging catheter will have a slightly different working distance, it will illuminate a different amount of the stroma, which may further impact the stroma measurements.

We present depth-resolved and distinct measurements for epithelium and stroma attenuation coefficient in vivo in the lateral and ventral tongue. An optical attenuation model has been previously suggested for oral cancer identification in a study of fresh ex vivo surgical samples from 14 oral cancer patients [33] where the average attenuation of squamous cell carcinoma was found to be 3.11 mm^−1^ and non-cancer margins 5.65 mm^−1^. This is comparable in trend to our overall attenuation coefficient measurement of 3.05 mm^−1^ in carcinoma although our contralateral measurements are lower (3.27 mm^−1^). Our findings suggest that focusing on the epithelial attenuation coefficient may provide better discrimination between lesions of different disease states than an overall attenuation coefficient.

### 4.4. Stratification Measurements

We anticipated that this feature would capture a decrease in stratification throughout disease progression as often, it becomes more challenging for an expert rater to distinguish epithelium and stroma. Instead, this appears to capture small connective tissue papillae (white arrows, Figure 4a(ii)) in the lower epithelium, which may be related to rete ridges or pegs, though co-registered histopathology is required to confirm this. This appears as small frequency ripples or pockmarks in healthy flat tissue but is dominated by larger changes in regions with high variability of epithelium depth. We believe these stratification measurements are promising, but perhaps, they would be better interpreted with texture analysis features rather than median intensity values.

### 4.5. Future Progression

Changes in stroma attenuation distinguishing progressors and non-progressors may point to differences in inflammatory cell infiltration and/or the extracellular matrix across the lesion and the contralateral in these two groups. Optical attenuation at 1310 nm has been shown to correlate positively with collagen content in ovarian tissue [48], so an increased stroma attenuation coefficient may point to collagen remodeling in progressors.

While these results are intriguing, this experiment is substantially limited by the sample size, with only four patients identified as future progressors with a mean of 45 months from the time of imaging to progression. While biopsy results are of the same lesion, we cannot be assured that they were from the same part of the lesion. We may have imaged an area of higher-grade lesion than the biopsy results at time of imaging, which could have resulted in cases being falsely labeled as ‘future progressors’. Further study with a larger sample size and correction for the confocal effect are required to understand this phenomenon. 

### 4.6. Future Directions

We see this device as having the best application for the assessment of large lesions in the oral cavity for biopsy site selection. Larger scale studies are required to confirm the relationship of these measurements to the disease status, to develop diagnostic criteria, to understand demographic and other pathologic confounders, and to generalize these findings to other patient cohorts. This device is able to access most parts of the oral cavity, and we have previously collected imaging from the buccal mucosa, floor of the mouth, gingiva, labial mucosa, lower lip, lateral dorsal and ventral tongue, and the vestibule [34]. In regions of bone-lined tissue, alternate catheter holders, such as the saliva ejector shown by Lee et al., may be used to position the device [30]. Multiple shorter scans can be acquired if tissue contact cannot be maintained over the entire area of interest. 

While this work focuses on endoscopic OCT, the measurements presented can be translated to galvanometer scanning approaches. The deep learning network is likely not generalizable beyond our endoscopic systems, but similar approaches could be taken to tailor an automated segmentation tool for other OCT devices. With a sufficient segmentation tool (or even with manual segmentation on select OCT frames), the same features could be calculated and examined.

If such a tool is to be used during clinical monitoring, rapid access to these measurements is required. The current analysis of whole-volume OCT through this approach is time-intensive; however, this is largely due to visualizing and saving data (longitudinal tiles, masks, etc.) throughout the process. With the device specifications described in the methods (Section 2.4), for a 5 cm long pullback (1024 × 512 × 5000 pixels before rescaling to 10 µm square pixels), the deep learning network predictions can be generated in 2.5 min, and once the masks are generated, all measurements can be calculated in 10 min. This may be improved via implementation in a language that is faster than MATLAB/Python such as C++.

## 5. Conclusions

We present a quantitative image processing analysis of endoscopic OCT of the oral cavity. This is a hypothesis-generating study intended to provide future direction for diagnostic criteria in oral OCT measurements: it is limited to a single-facility study with a small sample size examining many properties. While we have presented statistically significant findings, we note that we have not corrected for multiple comparisons and thus, larger studies are required to confirm these findings.

To briefly summarize, we demonstrate seven quantitative measurements of oral tissue (lateral or ventral tongue) in 40 patients with varying disease state using an endoscopic OCT catheter. These measurements are inclusive of morphology (epithelial depth, loss of epithelial–stromal boundary visualization), mean attenuation coefficients (overall, epithelium, stroma), and stratification of the attenuation coefficients (epithelial-stromal, intraepithelial stratification). 

We demonstrate the potential of these measurements to visually distinguish lesion margins, quantitatively differentiate the lesion from the contralateral, and quantitatively distinguish lesions clinically indicated for observation (benign lesions, mild, or moderate dysplasia) from intervention (severe dysplasia, carcinoma). We find that the median epithelial depth, loss of epithelial–stromal boundary visualization, and epithelial attenuation coefficient are significantly increased within lesions requiring intervention and are significantly different than their contralaterals for both groups. However, while the epithelium attenuation coefficient increases in lesions requiring intervention, the stromal attenuation coefficient decreases and dominates the measured overall attenuation coefficient. Both stratification features become closer to zero (lower contrast) in lesions requiring observation and demonstrate a significant difference between lesions requiring intervention and their contralateral. These changes can be visualized within each volume although there are confounders such as keratosis, which may appear similar to areas of lesion. 

In this dataset, the median epithelial depth of female contralaterals is lower than that of male patients. There are some additional suggestive positive correlations between age and the epithelial attenuation coefficient. There was no significant difference due to the presence of acanthosis or keratosis.

The overall and stroma attenuation coefficients demonstrate potential in distinguishing future progressors within the mild and moderate dysplasia groups. However, the sample size was substantially limited, and additional study is needed to draw conclusions about this effect. 

Last, we present two cases of repeatable measurements within one time point and five patients capturing some measured change over two time points, indicating that these tools may be able to capture longitudinal changes. As OCT is a label-free, non-ionizing imaging technique, this points to the potential for monitoring via imaging.

## Figures and Tables

**Table 1:** Summary of biomarkers.

Category	Biomarker	Description	Dimensionality
‘Morphologic’	Epithelium depth [μm]	Height of segmented epithelium region	2D en face image Range: 0 to ~2 mm
	Loss of epithelial–stromal boundary [%]	Percentage of loss over volume, excluding artifacts	Single value per volume Range: 0 to 100%
‘Attenuation’	Overall attenuation coefficient [mm^−1^]	Mean en face projection of attenuation coefficient over the entire depth of visualized tissue	3D data Range: 0 to ~10 mm^−1^
	Epithelium attenuation coefficient [mm^−1^]	Mean en face projection of attenuation coefficient over the segmented epithelium region	3D data Range: 0 to ~10 mm^−1^
	Stroma attenuation coefficient [mm^−1^]	Mean en face projection of attenuation coefficient over the visualized stroma region	3D data Range: 0 to ~10 mm^−1^
‘Stratification’	Epithelial–stromal stratification [a.u.]	$\frac{\mu_{epithelium}-\mu_{stroma}}{\mu_{epithelium}+\mu_{stroma}}$	2D en face image Range: −1 to 1 a.u.
	Intraepithelial stratification [a.u.]	$\frac{\mu_{upper\;epithelium}-\mu_{lower\;epithelium}}{\mu_{upper\;epithelium}+\mu_{lower\;epithelium}}$	2D en face image Range: −1 to 1 a.u.

**Table 2:** Dataset demographics for a single time point assessment of biomarkers.

Diagnosis	Lesion	Contralateral	Total	Males	Females
Only contralateral imaged	0	1	1	1	0
Benign	5	3	8	3	2
Mild dysplasia	8	7	15	3	5
Moderate dysplasia	10	8	18	4	6
Severe dysplasia	7	7	14	3	4
Carcinoma (squamous cell, verrucous)	9	5	14	6	3
Total	39 sites	31 sites	40 patients (70 sites)	20 patients (50%)	20 patients (50%)

**Table 3:** Dataset demographics for the assessment of future progression.

Diagnosis	Lesion	Contralateral	Total	Males	Females
Progressors	4	4	8	1	3
Non-progressors	11	11	22	5	6
Total	15 sites	15 sites	15 patients (33 sites)	6 patients (40%)	9 patients (60%)

**Table 4:** Dataset demographics for reproducibility/repeatability assessment.

Patient Number	Previous Biopsy (Time Difference) [Months]	Time Point 1			Time Difference [Months]	Time Point 2		
		Diagnosis	Lesion	Contra- Lateral		Diagnosis	Lesion	Contralateral
1	Mild dysplasia (unknown)	Mild dysplasia	1	1	5	Mild dysplasia	2	1
2	Moderate dysplasia (10)	Moderate dysplasia	2	1	2	Benign (hyperplastic candidiasis)	1	1
3	N/A	Moderate dysplasia	1	1	21	Moderate dysplasia	1	1
4	Moderate dysplasia (16)	Severe dysplasia	1	1	6	Severe dysplasia	1	1
5	Moderate dysplasia (13)	Verrucous carcinoma	1	1	6	Verrucous carcinoma	1	1

**Table 5:** Measurements of biomarkers across time points (contralateral samples) and repeated measurements of the lesion within a single time point. Data are presented as mean and absolute difference (|time point 2 − time point 1|) of the two measurements in that category.

		Morphologic Features		Mean Attenuation Coefficient			Stratification	
	Patient Number	Epithelium Depth	Loss of Epithelial–Stromal Boundary Visualization	Overall	Epithelium	Stroma	Epithelial- Stromal	Intraepithelial
		[µm]	[%]	[mm^−1^]	[mm^−1^]	[mm^−1^]	[a.u.]	[a.u.]
Contralateral (between time points)	1	240	2	3.05	1.22	3.71	−0.51	−0.21
		50	3	0.24	0.18	0.55	0.00	0.08
	2	150	0	3.59	1.15	4.21	−0.57	−0.12
		20	0	0.21	0.00	0.25	0.01	0.07
	3	160	0	3.76	1.12	4.47	−0.59	−0.15
		30	0	1.41	0.11	2.00	0.11	0.00
	4	120	0	4.25	0.95	4.95	−0.68	−0.14
		10	0	0.90	0.20	1.33	0.02	0.06
	5	220	0	3.60	1.36	4.52	−0.54	−0.23
		80	0	0.09	0.05	0.41	0.02	0.01
Lesion (single time point)	1	440	15	2.79	1.58	3.62	−0.41	−0.22
		40	2	0.04	0.21	0.09	0.05	0.04
	2	220	20	3.69	1.50	4.59	−0.53	−0.21
		50	29	0.15	0.39	0.00	0.08	0.01

## Data Availability

The data presented in this study are available on request from the corresponding author.

## Supplementary Materials

The following supporting information can be downloaded at: https://www.mdpi.com/article/10.3390/cancers16152751/s1, Table S1. Measurements of each feature per disease state. Table S2. Mean measurements of each feature per disease state and sex. Table S3. Measurements of other pathologic results in contralaterals. Table S4. Measurements against future progression status for mild and moderate dysplastic lesions.
