# Supplementary material for: Imaging Biomarkers of Oral Dysplasia and Carcinoma Measured with In Vivo Endoscopic Optical Coherence Tomography
Source: Cancers (Basel). 2024 Aug 2;16(15):2751. doi: 10.3390/cancers16152751 (PMC11311571; doi:10.3390/cancers16152751)
Supplement: Supplementary file 1 [file cancers-16-02751-s001.zip › cancers-3117820-supplementary.pdf]

cancers

MDPI

Supplemental Materials

Table S1. Measurements of each feature per disease state. Data is presented as mean, standard error, (range) of the median measurement of all volumes in that category.

			Morphologic Features		Mean Attenuation Coefficient			Stratification	
	**Sample size**	**Average age**	**Epithelium depth**	**Loss of epithelial–stromal boundary visualization**	**Overall**	**Epithelium**	**Stroma**	**Epithelial–Stromal**	**Intra-epithelial**
	#	[years]	[μm]	[%]	[mm^{-1}]	[mm^{-1}]	[mm^{-1}]	[a.u.]	[a.u.]
Contralateral	31	58	160<br>10<br>(120–260)	1<br>0<br>(0–4)	3.45<br>0.11<br>(2.26–4.17)	1.20<br>0.03<br>(0.99–1.74)	3.97<br>0.15<br>(2.43–5.64)	−0.52<br>0.02<br>− (0.69–0.27)	−0.18<br>0.01<br>−(0.28–0.10)
Benign	5	73	200<br>20<br>(140–280)	8<br>4<br>(1–18)	2.88<br>0.20<br>(2.30–3.44)	1.38<br>0.11<br>(1.13–1.66)	3.19<br>0.23<br>(2.60–3.85)	−0.41<br>0.03<br>−(0.50–0.32)	−0.17<br>0.02<br>−(0.22–0.11)
Mild dysplasia	8	47	210<br>10<br>(160–290)	5<br>3<br>(0–23)	3.62<br>0.14<br>(3.23–4.19)	1.36<br>0.09<br>(1.13–1.71)	4.35<br>0.18<br>(3.74–5.26)	−0.53<br>0.02<br>−(0.62–0.42)	−0.21<br>0.01<br>−(0.25–0.16)
Moderate dysplasia	10	58	320<br>70<br>(150–790)	22<br>10<br>(0–99)	3.44<br>0.19<br>(2.55–4.60)	1.80<br>0.21<br>(1.20–3.36)	4.01<br>0.25<br>(2.91–5.70)	−0.41<br>0.05<br>−(0.58–0.12)	−0.12<br>0.04<br>−(0.29–0.09)
Severe dysplasia	7	59	250<br>30<br>(180–360)	15<br>4<br>(0–27)	3.13<br>0.13<br>(2.61–3.71)	1.56<br>0.06<br>(1.25–1.71)	3.70<br>0.17<br>(3.15–4.53)	−0.43<br>0.03<br>−(0.57–0.35)	−0.16<br>0.03<br>−(0.25–0.08)
Carcinoma	9	56	560<br>80<br>(200–800)	77<br>9<br>(21–100)	3.05<br>0.12<br>(2.29–3.38)	2.48<br>0.22<br>(1.63–3.32)	3.56<br>0.18<br>(2.46–4.27)	−0.35<br>0.04<br>−(0.51–0.18)	−0.07<br>0.03<br>−(0.20–0.05)

Table S2. Mean measurements of each feature per disease state and sex. Data is presented as mean, standard error, (range) of the median measurement of all volumes in that category.

				**Morphologic Features**		**Mean Attenuation Coefficient**			**Stratification**	
	**Sex**	**Sample size**	**Average age**	**Epithelium depth**	**Loss of epithelial–stromal boundary visualization**	**Overall**	**Epithelium**	**Stroma**	**Epithelial–Stromal**	**Intra-epithelial**
	M<br>F	#	[years]	[μm]	[%]	[mm^{-1}]	[mm^{-1}]	[mm^{-1}]	[a.u.]	[a.u.]
Contralateral	M	14	56	180<br>10<br>(120–260)	1<br>0<br>(0–4)	3.29<br>0.17<br>(2.26–4.71)	1.21<br>0.04<br>(1.00–1.50)	3.79<br>0.22<br>(2.43–5.64)	−0.50<br>0.02<br>−(0.69–0.35)	−0.17<br>0.01<br>−(0.27–0.10)
	F	17	60	140<br>10<br>(120–200)	0<br>0<br>(0–1)	3.58<br>0.14<br>(2.36–4.61)	1.19<br>0.05<br>(0.99–1.74)	4.12<br>0.20<br>(2.45–5.51)	−0.54<br>0.02<br>−(0.68–0.27)	−0.19<br>0.01<br>−(0.28–0.11)
Benign	M	3	79	220<br>30<br>(190–280)	13<br>5<br>(3–18)	2.76<br>0.27<br>(2.30–3.24)	1.49<br>0.14<br>(1.21–1.66)	3.07<br>0.28<br>(2.60–3.58)	−0.37<br>0.03<br>−(0.40–0.32)	−0.18<br>0.02<br>−(0.22–0.14)
	F	2	65	150<br>10<br>(140–170)	2<br>1<br>(1–3)	3.06<br>0.38<br>(2.69–3.44)	1.21<br>0.07<br>(1.13–1.28)	3.38<br>0.48<br>(2.90–3.85)	−0.47<br>0.03<br>−(0.50–0.45)	−0.17<br>0.05<br>−(0.22–0.11)
Mild dysplasia	M	3	52	240<br>30<br>(180–290)	4<br>4<br>(0–11)	3.37<br>0.09<br>(3.23–3.53)	1.33<br>0.15<br>(1.15–1.63)	4.09<br>0.11<br>(3.89–4.24)	−0.52<br>0.05<br>−(0.57–0.42)	−0.20<br>0.03<br>−(0.25–0.17)
	F	5	44	200<br>10<br>(163–230)	6<br>4<br>(0–23)	3.76<br>0.19<br>(3.24–4.19)	1.37<br>0.13<br>(1.13–1.71)	4.51<br>0.27<br>(3.74–5.26)	−0.54<br>0.02<br>−(0.62–0.50)	−0.21<br>0.01<br>−(0.24–0.16)
Moderate dysplasia	M	4	64	390<br>140<br>(230–790)	35<br>22<br>(3–99)	3.18<br>0.15<br>(2.86–3.49)	2.06<br>0.43<br>(1.55–3.63)	3.64<br>0.18<br>(3.40–4.17)	−0.33<br>0.07<br>−(0.43–0.12)	−0.07<br>0.06<br>−(0.18–0.09)
	F	6	54	280<br>90<br>(150–710)	13<br>9<br>(0–52)	3.60<br>0.28<br>(2.55–4.60)	1.62<br>0.21<br>(1.20–2.28)	4.26<br>0.39<br>(2.91–5.70)	−0.46<br>0.07<br>−(0.58–0.22)	−0.15<br>0.04<br>−(0.29–0.02)
Severe dysplasia	M	3	52	220<br>20<br>(180–240)	12<br>7<br>(0–26)	3.40<br>0.17<br>(3.12–3.71)	1.51<br>0.14<br>(1.25–1.71)	4.05<br>0.29<br>(3.52–4.53)	−0.47<br>0.06<br>−(0.57–0.37)	−0.15<br>0.04<br>−(0.20–0.08)
	F	4	65	280<br>40<br>(210–360)	17<br>5<br>(6–27)	2.93<br>0.11<br>(2.61–3.13)	1.60<br>0.05<br>(1.48–1.70)	3.44<br>0.11<br>(3.15–3.65)	−0.40<br>0.02<br>−(0.44–0.35)	−0.17<br>0.04<br>−(0.25–0.08)
Carcinoma	M	6	47	510<br>120<br>(200–800)	74<br>13<br>(21–100)	3.05<br>0.16<br>(2.29–3.31)	2.28<br>0.27<br>(1.63–3.17)	3.60<br>0.27<br>(2.46–4.27)	−0.37<br>0.05<br>−(0.51–0.18)	−0.09<br>0.05<br>−(0.20–0.05)
	F	3	76	670<br>60<br>(600–900)	82<br>11<br>(60–95)	3.06<br>0.23<br>(2.62–3.38)	2.89<br>0.25<br>(2.44–3.32)	3.48<br>0.11<br>(3.26–3.64)	−0.31<br>0.03<br>−(0.36–0.24)	−0.03<br>0.01<br>−(0.05–0.02)

Table S3. Measurements of other pathologic results in contralaterals. Data is presented as mean, standard error, (range) of the median measurement of all volumes in that category.

			Morphologic Features		Mean Attenuation Coefficient			Stratification	
	**Sample size**	**Average age**	**Epithelium depth**	**Loss of epithelial–stromal boundary visualization**	**Overall**	**Epithelium**	**Stroma**	**Epithelial–Stromal**	**Intra-epithelial**
	#	[years]	[μm]	[%]	[mm⁻¹]	[mm⁻¹]	[mm⁻¹]	[a.u.]	[a.u.]
Acanthosis	13	57	170<br>10<br>(120–260)	1<br>0<br>(0–4)	3.42<br>0.16<br>(2.37–4.57)	1.18<br>0.05<br>(0.99–1.50)	3.96<br>0.21<br>(2.56–5.42)	−0.53<br>0.02<br>−(0.68–0.36)	−0.19<br>0.01<br>−(0.27–0.13)
No Acanthosis	18	58	150<br>10<br>(120–260)	1<br>0<br>(0–4)	3.44<br>0.16<br>(2.26–4.71)	1.22<br>0.04<br>(1.02–1.74)	3.95<br>0.22<br>(2.43–5.64)	−0.52<br>0.03<br>−(0.69–0.27)	−0.17<br>0.01<br>−(0.28–0.10)
Keratosis	15	55	160<br>10<br>(120–260)	1<br>0<br>(0–4)	3.63<br>0.15<br>(2.92–4.71)	1.17<br>0.04<br>(0.99–1.50)	4.23<br>0.19<br>(3.24–5.64)	−0.56<br>0.02<br>−(0.69–0.44)	−0.19<br>0.01<br>−(0.27–0.11)
No keratosis	16	60	160<br>10<br>(120–200)	1<br>0<br>(0–4)	3.24<br>0.16<br>(2.26–4.21)	1.23<br>0.05<br>(1.02–1.74)	3.68<br>0.21<br>(2.43–4.95)	−0.49<br>0.03<br>−(0.63–0.27)	−0.17<br>0.01<br>−(0.28–0.10)

Table S4. Measurements against future progression status for mild and moderate dysplastic lesions (P = progressors, NP = non−progressors). Data is presented as mean, standard error, (range) of the median measurement of all volumes in that category. 'Difference' is the pairwise subtraction of contralateral median value from lesion median value for each volume.

				**Morphologic Features**		**Mean Attenuation Coefficient**			**Stratification**	
	**Progression status**	**Sample size**	**Average age**	**Epithelium depth**	**Loss of epithelial–stromal boundary visualization**	**Overall**	**Epithelium**	**Stroma**	**Epithelial–Stromal**	**Intra–epithelial**
	P NP	#	[years]	[µm]	[%]	[mm^{-1}]	[mm^{-1}]	[mm^{-1}]	[a.u.]	[a.u.]
Contralateral		4		150	0	3.32	1.09	3.77	−0.55	−0.18
	P	(1 D1,	55	20	0	0.16	0.07	0.18	0.03	0.02
		3 D2)		(120−200)	(0)	(2.99−3.60)	(0.99−1.29)	(3.45−4.10)	−(0.60−0.46)	−(0.21−0.14)
		11		160	1	3.81	1.18	4.47	−0.58	−0.18
	NP	(6 D1,	54	10	0	0.16	0.05	0.20	0.02	0.01
		5 D2)		(120−260)	(0−3)	(3.05−4.61)	(1.00−1.50)	(3.47−5.51)	−(0.68−0.44)	−(0.24−0.13)
Lesion		4		190	7	3.45	1.32	4.04	−0.51	−0.18
	P	(1 D1,	55	20	5	0.19	0.13	0.24	0.04	0.01
		3 D2)		(160−230)	(0−21)	(3.02−3.86)	(1.13−1.70)	(3.56−4.59)	−(0.56−0.38)	−(0.21−0.16)
		11		220	5	3.60	1.47	4.35	−0.50	−0.18
	NP	(6 D1,	54	20	2	0.15	0.10	0.21	0.03	0.02
		5 D2)		(150−290)	(0−22)	(2.86−4.60)	(1.13−2.25)	(3.40−5.70)	−(0.62−0.28)	−(0.29−0.03)
Difference		4		40	7	0.13	0.24	0.27	0.04	0.005
	P	(1 D1,	55	20	5	0.19	0.13	0.24	0.04	0.01
		3 D2)		(30−50)	(0−21)	(0.03−0.27)	(0.13−0.41)	(0.11−0.51)	(0.01−0.07)	(−0.04−0.05)
		11		60	5	−0.21	0.30	−0.12	0.08	0.003
	NP	(6 D1,	54	20	2	0.15	0.10	0.21	0.03	0.02
		5 D2)		(−80−140)	(−3−22)	(−0.60−0.36)	(−0.12−1.15)	(−0.77−0.26)	(−0.05−0.32)	(−0.09−0.18)
